# Earthworm coelomocyte extracellular traps: structural and functional similarities with neutrophil NETs

**DOI:** 10.1007/s00441-018-2787-0

**Published:** 2018-02-05

**Authors:** Joanna Homa

**Affiliations:** Department of Evolutionary Immunology, Institute of Zoology and Biomedical Research, Jagiellonian University, Gronostajowa 9, 30-387 Krakow, Poland

**Keywords:** Coelomocytes, Amoebocytes, Eleocytes, Extracellular traps, Histones

## Abstract

Invertebrate immunity is associated with natural mechanisms that include cellular and humoral elements, similar to those that play a role in vertebrate innate immune responses. Formation of extracellular traps (ETs) is a newly discovered mechanism to combat pathogens, operating not only in vertebrate leucocytes but also in invertebrate immune cells. The ET components include extracellular DNA (exDNA), antimicrobial proteins and histones. Formation of mammalian ETs depends on enzymes such as neutrophil elastase, myeloperoxidase, the citrullination of histones and protease activity. It was confirmed that coelomocytes—immunocompetent cells of the earthworm *Eisenia andrei*—are also able to release ETs in a protease-dependent manner, dependent or independent of the formation of reactive oxygen species and rearrangement of the cell cytoskeleton. Similar to vertebrate leukocytes (e.g., neutrophil), coelomocytes are responsible for many immune functions like phagocytosis, cytotoxicity and secretion of humoral factors. ETs formed by coelomocyte analogues to neutrophil ETs consist of exDNA, histone H3 and attached to these structures proteins, e.g., heat shock proteins HSP27. The latter fact confirms that mechanisms of ET release are conserved in evolution. The study on Annelida adds this animal group to the list of invertebrates capable of ET release, but most importantly provides insides into innate mechanisms of ET formation in lower animal taxa.

## Introduction

The earthworm immune response demonstrates a number of structural and functional similarities to the innate immune system of vertebrates. In invertebrates with a secondary body cavity (e.g., Annelids), coelomic fluid is rich in many proteins (lysozyme, fetidins, lysine protease) and specific cells, i.e. coelomocytes, which can be classified as amoebocytes and eleocytes (Bilej et al. 2010). On the other hand, in invertebrates that have an open circulatory system, such as arthropods (insects, crustaceans) and molluscs, hemocytes are responsible for phagocytosis and cytotoxicity. Hemocytes can be further subdivided into hyaline hemocytes and granulocytes. These cells, together with numerous humoral components (e.g., cecropins, defensins, proteases) are present in the hemolymph (Söderhäll 2010). Regardless of the adopted cell names of coelomocytes and hemocytes, their killing mechanisms are similar to each other and pathogen destruction is based on phagocytosis, enzyme activation (e.g., lysozyme), and formation of reactive oxygen species (ROS) and antimicrobial proteins (e.g., defensins) (Bilej et al. 2010; Söderhäll 2010). Recent papers also confirm the possibility that invertebrate phagocytes are capable to produce extracellular traps (ETs) (e.g., Homa et al. 2016a; Robb et al. 2014).

### Anatomy of the earthworm immune system and immune effector mechanisms

**Fig. 1:**
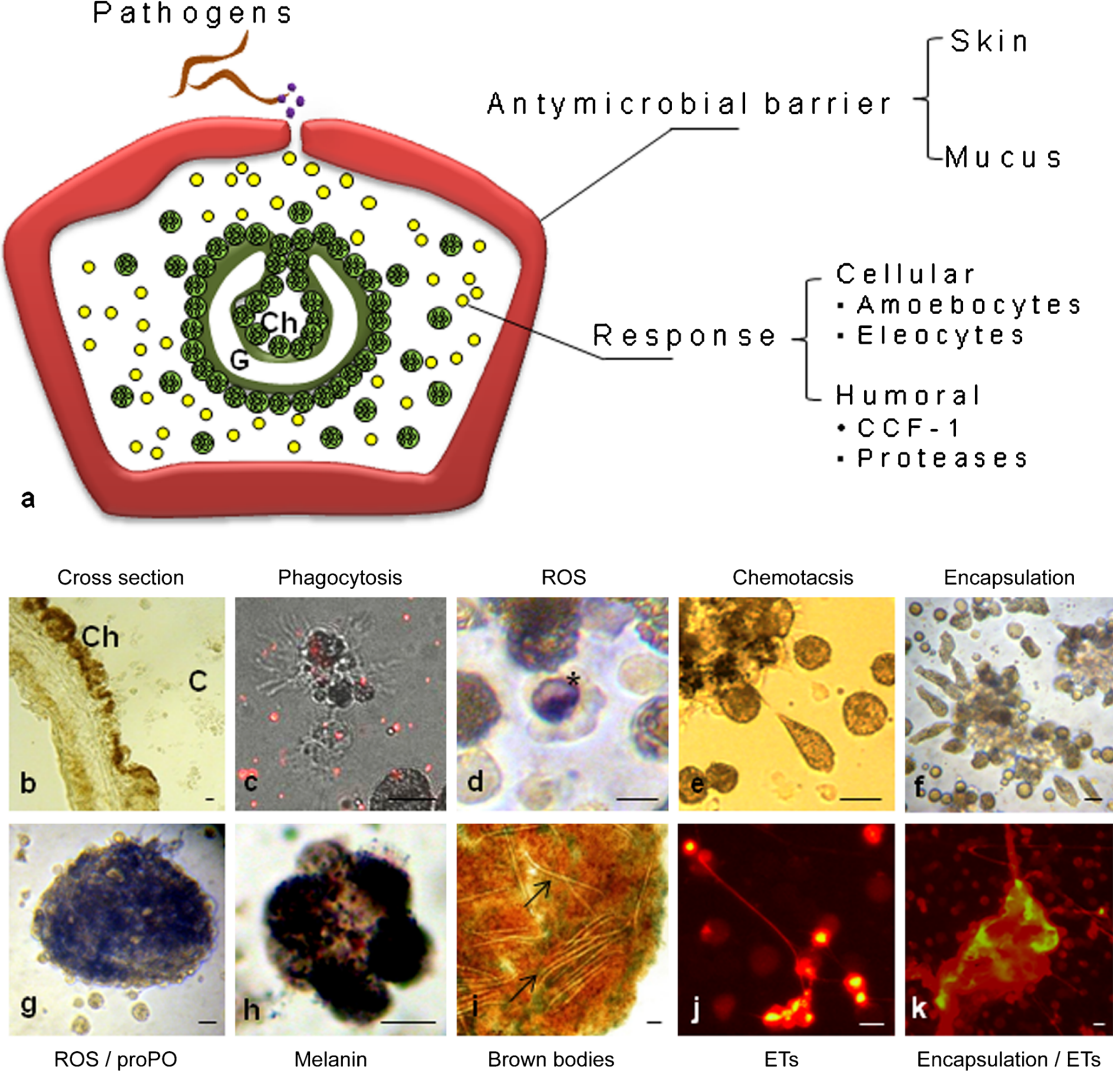
Anatomy of the earthworm (*Eisenia andrei*) immune system and immune effector mechanisms. **a** Cross-section of earthworm and their elements of immune system: surrounding the gut (*G*), chloragogen tissue (*Ch*) and free-floating coelomocytes; amoebocytes and free eleocytes derived from chloragogen tissue. Representative images of coelomocytes’ basic immune reactions: **b** cross-section with visible chloragogen tissue (*Ch*) and in coelom cavity free coelomocytes (*C*), **c** phagocytosis, **d** ROS production, cell containing dark blue NBT formazan deposits (*), **e** moving cells – chemotaxis, **f** encapsulation, **g** ROS and proPO activation in the formed kapsule and **h** melanin synthesis (dark deposits) which finally leading to brown bodies formation, e.g., **i** nematodes closure, visible inside the capsule (*arrow*), **j** the latest mechanism of coelomocytes response, production of extracellular traps (*ETs*) and **k** joint action of encapsulation and ETs formation process (Sytox orange staining). *Scale bar* 25 μm

The earthworms are protostomian animals possessing true coelom cavity filled with coelomic fluid that not only forms a stable hydrostatic skeleton but also includes many cells of the immune system, coelomocytes and humoral factors (Bilej et al. 2010; Cooper et al. 2002). The coelomocytes originate in the mesenchymal lining of the cavity (Bilej et al. 2010) and are the primary immune cells of earthworms. In simplified nomenclature, coelomocytes are divided into amoebocytes (hyaline and granular) and cells derived from chloragogen tissue surrounding the gut, called eleocytes/chloragocytes (Kurek et al. 2007; Bilej et al. 2010) (Fig. 1a, b). Taking into account physical parameters measured by flow cytometry, small and large coelomocytes with different functional characteristics may be distinguished (Cooper et al. 1995, 2002; Cossarizza et al. 1996; Quaglino et al. 1996). In turn, Engelmann and coworkers identified using flow cytometry three different populations of coelomocytes: (1) R1 – granular coelomocytes, (2) R2 – hyaline cells, and (3) R3 – chloragocytes/eleocytes (Engelmann et al. 2004, 2005). Moreover, in some older classifications based on cytomorphology and cytochemistry, the coelomocytes of the annelid (e.g., *Eisenia fetida*) were divided into four major categories: acidophils, basophils, chloragocytes cells, and neutrophils (Stein and Cooper 1978). Amoebocytes are involved in the immune response including phagocytosis (Valembois and Lassègues 1995), ROS production (Homa et al. 2013, 2016b), and cytotoxicity (NK cell-like activity) (Cossarizza et al. 1996). They also express Toll-like receptors (TLRs) (Škanta et al. 2013; Fjøsne et al. 2015). It is known that antimicrobial AMP-like protein of the neutrophil granule content in the function are similar to lipopolysaccharide-binding protein (LBP) and bacterial permeability-increasing protein (BPI) (Wiesner and Vilcinskas 2010). Similarly to neutrophils, coelomocytes of the earthworm *Eisenia andrei* express genes uncoding for at least two conserved domains (*Ealbp/bpi* and *ccf*) with the ability to bind lipopolysaccharide (LPS). They differ in their tissue expression and share homology with LBP/BPI family (Škanta et al. 2016). According to the authors, the up-regulation of mRNA level of *Ealbp/bpi* after bacterial infection suggests their significant role in earthworm immune defense (Škanta et al. 2016).

On the other hand, eleocytes synthesize and release humoral factors, such as agglutinins and opsonins (Bilej et al. 2010). Important antimicrobial peptides (AMPs), belonging to two structurally distinct classes, known as the defensins and the cathelicidins, are mainly produced by vertebrate neutrophils (Wiesner and Vilcinskas 2010). Several authors have demonstrated that earthworm innate immunity also depends on coelomocytes that synthesize and secrete humoral antimicrobial molecules (e.g., lysenin, fetidin, coelomic cytolytic factor 1, CCF-1) (e.g., Bilej et al. 2000, 2001, 2010; Engelmann et al. 2005). Among subpopulations of coelomocytes, lysenin is mainly produced by chloragocytes and its expression can be modulated by Gram-positive bacterial exposure (Opper et al. 2013). In turn, CCF-1 is localized in the cells of chloragogenous tissue adjunct to the gut wall and in the translucent free large coelomocytes, i.e. in cells with macrophage-like function (Bilej et al. 1998). Among others, CCF-1 is involved in pathogen recognition and leads to its immobilization (Bilej et al. 2001). In addition, eleocytes, derived from chloragogen tissue, are responsible for maintaining the constant pH of coelomic fluid and storage of glycogen and lipids (Affar et al. 1998; Fischer and Molnár 1992). Moreover, eleocyte granules store riboflavin (B2 vitamin) (Plytycz et al. 2006). In the earthworm coelom cavity, numerous enzymes such as proteases are also present. The proteases exert antimicrobial effects and take part in the activation of the prophenoloxidase system (pro-PO) (Valembois et al. 1994). The final stage of pro-PO activation is melanization and elimination of pathogens (e.g., nematodes) (Fig. 1g–i).

Earthworms, during their defense against pathogens, use several elementary mechanisms. Phagocytosis by coelomocytes, similarly to that of vertebrates, can be modulated by humoral components, opsonins, which coat the particle and thus promote its phagocytosis. Moreover, they are capable of ROS and nitric oxide (NO) production (Homa et al. 2013; Bernard et al. 2015; Homa et al. 2016b; Valembois and Lassègues 1995). Furthermore, coelomocytes have a variety of defense mechanisms to resist the harmful side effects of ROS. They include expression of superoxide dismutase (SOD) which catalyzes the conversion of superoxide into hydrogen peroxide and oxygen, as well as glutathione peroxidases and catalases, which then degrade hydrogen peroxide (Homa et al. 2016b; Saint-Denis et al. 1998).

The above-mentioned molecules are key factors in the process of chemotaxis, phagocytosis and encapsulation, i.e. closing the pathogens inside structures called “brown bodies” (Bilej et al. 2010; Valembois et al. 1992) (Fig. 1c–i). Encapsulation is a cellular immune response used against pathogens that are too large to be phagocytosed (Valembois et al. 1994). “Brown bodies” are gradually pushed into the posterior parts of the earthworm body, and finally disposed with segments through the natural amputation called autotomy (Bilej et al. 2010).

In many groups of invertebrates, the pro-PO, an element of the humoral innate immune system, is the first line of defense in the fight against pathogens. Phenoloxidase (PO) is a part of a complex system of pattern recognition, made of proteinases and proteinase inhibitors, constituting the so-called prophenoloxidase-activating system (Söderhäll 2010). This innate immune reaction provides toxic quinone substances and other short-lived reaction intermediates involved in the formation of more long-lived products, such as melanin, that physically encapsulate pathogens (Valembois et al. 1992, 1994). Recent evidence also strongly implies that the melanization cascade provides, or is intimately associated with, the appearance of factors stimulating cellular defense by aiding phagocytosis. In annelids, the pro-PO system is strictly involved in encapsulation and the formation of brown bodies, in which melanin and lipofuscin are synthesized. Therefore, it is not surprising that several studies have unequivocally shown the importance of the melanization reaction for the outcome of several specific pathogen–host encounters, including bacterial infections.

### Extracellular trap production

Since the discovery of ETs, the results of research conducted on vertebrate cells have added much information on both the components of ETs and the mechanisms necessary to initiate their formation (Brinkmann et al. 2004; Neeli et al. 2009; Papayannopoulos et al. 2010; Kolaczkowska et al. 2015). The phenomenon of creating ETs was first described for mammalian neutrophils (Brinkmann et al. 2004). The authors concluded that, upon stimulation with Gram-positive (*Staphylococcus aureus*) or Gram-negative (*Salmonella typhimurium* and *Shigella flexneri*) bacteria, as well as under the influence of phorbol 12-myristate 13-acetate (PMA), LPS and interleukin-8 (IL-8) neutrophils are able to produce ETs, so-called neutrophil ETs (NETs), in which DNA and cytoplasmic granule factors are contained. The following years brought reports on the ability to also create ETs by other populations of mammalian leukocytes, i.e., monocytes/macrophages, eosinophils, and mast cells (Chow et al. 2010; Yousefi et al. 2008) in mice (Kolaczkowska et al. 2015), sheep and cattle (Yildiz et al. 2017), as well as by other non-mammalian vertebrate neutrophils and macrophages, e.g., teleost fish (Pijanowski et al. 2013) and chicken (Chuammitri et al. 2017). The production of ETs is important in the defense against pathogens, but there is still no clear evaluation of the whole range of consequences of their activation. Although 13 years has passed by since the discovery of ET structures, the number of reports on ETs in invertebrates is still limited. To date, it has been found that ETs are produced by the hemocytes of shrimps (Ng et al. 2013, 2015; Koiwai et al. 2016), crab (*Carcinus maenas*) (Robb et al. 2014), oyster (*Crassostrea gigas*) (Poirier et al. 2014), gastropod slug species (*Arion lusitanicus* and *Limax maximus*), and snail (*Achatina fulica*) (Lange et al. 2017). The latest reports indicate that the cells of simpler organisms, e.g., the social amoeba (*Dictyostelium discoideum*), also have an ability to release extracellular DNA with the formation of structures similar to NETs (Zhang et al. 2016; Zhang and Soldati 2016). Earthworm coelomocytes show a similar mechanism (Homa et al. 2016a).

In some studies of the structure of ETs released from invertebrate immunocompetent cells, only the presence of extracellular DNA (extDNA) was found after cell immunological stimulation (Koiwai et al. 2016). Other studies have revealed that histones (Ng et al. 2013; Robb et al. 2014; Homa et al. 2016a), hsp 27 (Homa et al. 2016a) and c-type lysozyme (Koiwai et al. 2016) are also attached to extDNA. The most detailed characteristic of ETs was revealed in shrimp hemocytes (Ng et al. 2013, 2015). They demonstrated that *E. coli* can be captured by ETs and that histone H1 proteins colocalized with DNA fibers. A very interesting process of ET formation was also found in social amoeba (Zhang et al. 2016; Zhang and Soldati 2016). During the emergence of multicellularity, these animals developed a primitive immune system in the form of a dedicated set of specialized phagocytic cells including cells (Sentinel cells) which release ET structures.

Based on knowledge gained through research on vertebrate cells, it is known that the mechanism of ET formation consists of several basic steps, as follows: (1) production of ROS and (2) the transport of proteases, including neutrophil elastase responsible for the chromatin decondensation, from cytoplasmic granules to the cell nucleus (Papayannopoulos and Zychlinsky 2009). The next step of the ET formation is the citrullination of histones, and, finally, generation of ETs, which means throwing unfolded DNA together with granule components out of the cell (Brinkmann et al. 2004; Kolaczkowska et al. 2015). In general, the proteins attached to neutrophil ETs include histones, proteases (e.g., neutrophil elastase, cathepsin G), oxidative enzymes (e.g., myeloperoxidase, MPO) and antimicrobial proteins such as lactoferrin (Goldmann and Medina 2013; Vorobjeva and Pinegin 2014). It should be underlined that histones are the main protein components of chromatin that compact, help condensate DNA, and possess antimicrobial properties (Brinkmann et al. 2004). Moreover, recent research suggests that the underlying structure of NETs is considerably organized and that part of their protein content plays an important role in maintaining their mesh architecture (Pires et al. 2016).

**Fig. 2:**
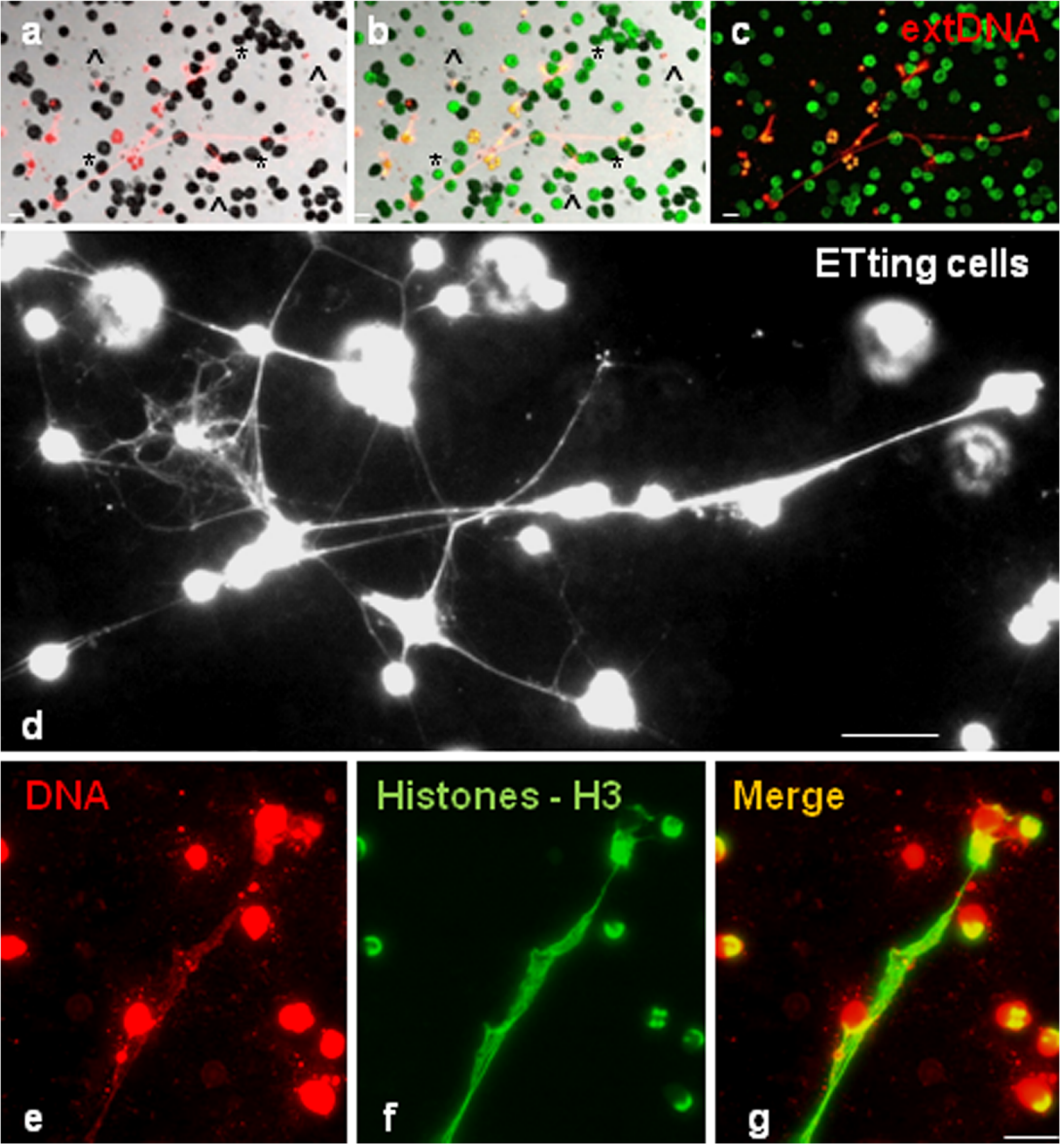
Earthworm (*Eisenia andrei*) coelomocytes form extracellular traps (ETs) composed of extracellular DNA (*extDNA*) and histones. **a** Representative images of live coelomocytes that released ETs or are in a process of their release (*ETting*). Coelomocytes retrieved from *E. andrei* were seated in slide chambers and stimulated with PMA and, after 24 h, Sytox orange was added to stain the extDNA. **b** Autofluorescent eleocytes (*, green fluorecscence is derived from riboflavin) and amoebocytes (^), **c** some coelomocytes in a process of extruding their DNA (ET). **d** Representative images of immunofluorescence staining of ETs released by *E. andrei* coelomocytes collected from earthworms treated for 24 h with bacteria *X. bovienii*. Retrieved coelomocytes were seated in slide chambers and the immunostaining was performed after 24 h; additionally, **e** Sytox orange was used to counter-stain extDNA. **f**,** g **Immunostaining with specific antibodies revealed that extDNA (*red*) is decorated with histones 3 (*H3*, *green*). *Scale bar* 25 μm

In studies on earthworm coelomocytes, we demonstrated the appearance of NET-like structures (Fig. 1j, k) as a result of coelomocyte stimulation with LPS, zymosan, PMA, as well as *Micrococcus lysodeikus* and *Xenorhabdus bovienii* (symbiotic bacteria inhabiting nematodes). Moreover, it was revealed that the coelomocyte ETs are built, among others, of nuclear DNA, H3 histones (Fig. 2a–g) and conserved heat shock proteins HSP27 (Homa et al. 2016a). However, it should be mentioned that the lack of specific antibodies makes studies of invertebrate ETs very difficult.

The results indicate a strong similarity of invertebrate ETs to originally described ETs formed by vertebrate neutrophils. Moreover, both in studies of vertebrate and invertebrate ETs, inhibitors of proteases, neutrophil elastase and NADPH oxidase were used to reveal the mechanisms responsible for ET triggering. Serine proteases, including elastase-like protease called earthworm fibrynolytic enzyme (EFE), have also been described in Annelida (Zhao et al. 2007). EFE degrades fibrinogen, elastin and fibrin, but also partially converts plasminogen into active plasmin (Zhao et al. 2007). In our experiments on earthworm ETs, we found that protease inhibitors including serine proteases and elastase inhibit ET formation while the inhibitors of autophagy and the inhibitors of apoptosis-promoting caspases did not hinder this process (Homa et al. 2016a). Surprisingly, it was shown that NET formation in human neutrophils is dependent on autophagy (Remijsen et al. 2011a).

Intriguingly, Pieterse et al. (2016) observed that, in whole blood cultures ex vivo or in vitro in the presence of platelets, all LPS serotypes induced “vital” NET formation. This platelet-dependent release of NETs occurred rapidly without neutrophil cell death and was independent of ROS formation and autophagy but required platelet TLR4- and CD62P-dependent platelet–neutrophil interactions. Nevertheless, the inhibition of ROS (with DPI) or autophagy (with wortmannin) did not influence “vital” NETosis induced by LPS-O111 (Pieterse et al. 2016). Moreover, it was recently demonstrated that LPS-activated platelets induce “vital” NETosis during sepsis (Ma and Kubes 2008; Yipp and Kubes 2013). This form of NET release is fundamentally different from “suicidal” NETosis; hence, “vital” NETosis occurs much faster, is not dependent on autophagy or ROS, and is not associated with direct lytic cell death. In contrast to apoptotic cells, NET formation involved different mechanisms without signals such as phosphatidyl serine before plasma membrane disruption (Remijsen et al. 2011a). Moreover, caspase activity is only detected during spontaneous neutrophil apoptosis, but not during, e.g., PMA-induced NETosis (Remijsen et al. 2011b). Furthermore, in coelomocytes, the NADPH oxidase inhibitor, suppressing the respiratory burst, exerted an inhibitory effect on the ETs formation in cells stimulated with PMA but not upon stimulation with bacteria. These results have confirmed earlier observations in vertebrates (Kolaczkowska et al. 2015; Pijanowski et al. 2013) that the production of ETs is not always ROS-dependent.

As mentioned above, the ETs contain histones, but, interestingly, parts of them are citrullinated histones. It is known that the packing of nuclear chromatin is associated with the presence of histones, and its decondensation is partially dependent on an appropriate modification of these conservative proteins. There is also evidence that histones are subject to a number of post-translational modifications, from which citrullination (deimination of guanidine residues in arginines) in histones is essential for NET formation. In vertebrates, PAD4 (peptidylarginine deiminase 4) is the enzyme responsible for histone citrullination (Rohrbach et al. 2012). As, to date, PAD4 has not been detected in lower organisms (Bachand 2007), the mode of ET-contained histone citrullination still remains unclear. Surprisingly, in our recent study (Homa et al. 2016a), an inhibitory effect of a well-known PAD4 inhibitor (Cl-amidine) on ET formation in earthworm coelomocytes, as well as the presence of citrullinated H3 histones within the ETs, was found. These results suggest the potential to carry out the process of H3 histone citrullination in earthworms, and the possibility of the presence of an enzyme that plays a similar role and shows susceptibility to the standard PAD4 inhibitor. To support this conclusion, it is worth noting that the mechanism of the ET formation in invertebrates, including earthworms, exhibits many similarities with the mechanism described in vertebrates (Table 1). As mention before, these similarities can be found even in the presence and activity of serine proteases, production of ROS and the activity of antioxidant enzymes.

**Table 1:** Summary of similarities between earthworm coelomocytes extracellular traps and vertebrate neutrophil extracellular traps

Neutrophil extracellular traps^a^	Coelomocytes extracellular traps^b^
extDNA	extDNA
Histones	Histones (H3)
Neutrophil elastase NE	Elastase–like proteases
Myeloperoxidase MPO	Proteases
PAD4/Cytrulination	PAD4 - not detected in invertebrates/cytrulination?
Cytoplasmic/granular proteins	Cytoplasmic/granular proteins
ROS-dependent or non-dependent	ROS–dependent or non-dependent

^a^Brinkmann et al. 2004; Papayannopoulos and Zychlinsky 2009

^b^Homa et al. 2016a

Studies conducted to date have allowed scientists to identify considerable similarities between the formation and composition of ETs in earthworms and structures formed by vertebrate neutrophils. It should be noted, however, that many aspects related to the invertebrate ETs have not yet been verified.

One more question which has not been revealed until now is the involvement of ETs in the process of the eradication of larger pathogens. The immune system of both vertebrates and invertebrates controls pathogens of varying sizes, ranging from small viruses and bacteria to fungi and parasites. Large pathogens (e.g., parasites) avoid phagocytosis and therefore can be difficult to remove (Branzk et al. 2014). As explained in the previous section, encapsulation and formation of brown bodies play a paramount role in removing bigger pathogens (e.g., nematodes), and eliminating bacteria or the cells contained in the structure of capsule (Valembois et al. 1994). Within such aggregates, activated coelomocytes generate ROS, and activate the proPO system. The latter is dependent on the action of proteases. In turn, melanin deposition occurs within the borders of brown bodies. The melanin is involved in the separation of pathogens from the coelom. The identity of mechanisms/ molecules involved in the formation of brown bodies and ETs suggest that these are connected processes. And, indeed, it was found that the extracellular DNA may facilitate the agglomeration of cells and formation of brown bodies (Homa et al. 2016a).

## Life is all about evolution: from ETs to NETs

The earthworms immune system when stimulated shows phagocytosis, encapsulation, agglutination, opsonization, clotting and lysis. The list of earthworm defense mechanisms demonstrated that coelomocytes can also form ETs which successfully trap bacteria. Similar to vertebrates, earthworm ETs are DNase- and heparin-sensitive. ETs formation by coelomocytes depends on protease activity but is independent of coelomocyte apoptosis and NADPH oxidase-independent in the case of bacteria-induced ETs, in contrast to ROS-dependent ET formation upon PMA-stimulation. Moreover, coelomocyte ETs trap bacteria and are involved in the formation of cell aggregates (Homa et al. 2016a). Furthermore, the results obtained on Sentinel cells of social amoebae (Zhang et al. 2016) are strong evidence that DNA-based cell-intrinsic defense mechanisms emerged much earlier than thought, about 1.3 billion years ago (Zhang and Soldati 2016). Interestingly, in plants, upon infection, specialized cells on the surface of a root also release their chromatin in a process that requires ROS production (Hawes et al. 2011). These NET-like structures have a defense function, as degrading them with DNases makes the plant more susceptible to fungal infections.

In invertebrates, the released chromatin participates in defense not only by ensnaring microorganisms and also by externalizing antibacterial histones together with other coelomocyte-/haemocyte-derived defense factors, but, crucially, also provides the scaffold on which intact cells assemble during encapsulation; a response that sequesters and kills potential pathogens infecting the body cavity (Robb et al. 2014).

What is the ET/NET function, immobilization or active killing? The antimicrobial activity of ETs is likely the result a combination of the components, and their effects are enhanced by the high local concentrations achieved in the NET structure. Lastly, antibodies against histones prevent NET-mediated killing of various microorganisms (Brinkmann et al. 2004), underlining the finding that these abundant proteins kill microbes very efficiently. Histones are indispensable for eukaryotic and archaeal life. Histones are highly conserved through evolution, form the basic unit of the chromatin, the nucleosome, and have been intensively studied and are well characterized (Thatcher and Gorovsky 1994; Kornberg and Lorch 1999). In mammals, extranuclear histones are found in the cytoplasm and on the surface of cells and are released abundantly in NETs (Urban et al. 2009; Brinkmann and Zychlinsky 2012). Invertebrate histones also show antimicrobial activity against a wide range of microorganisms: bacteria and parasites in vitro and in vivo and have the ability to bind bacterial lipopolysaccharide and other pathogen-associated molecules (Nikapitiya et al. 2013). For example, a mix of core histone proteins H2A, H2B, H3, and H4, isolated from the hemocytes of the Pacific white shrimp, have antimicrobial activity against *Micrococcus luteus* (Patat et al. 2004).

The expulsion of chromatin as a weapon might well be an ancient tool conserved in evolution in the form of ETs. Exploring how ETs are made and testing their relevance during disease and in health could enhance our understanding of this novel aspect of immunity. ETs could, on the host side, help organisms survive in an environment where predation and parasitism by microbes are a threat. However, ETs drive the evolutionary selection of more pathogenic strains of microorganisms (Brinkmann and Zychlinsky 2012).

Such a tactic of fight pathogens has always been needed, even in the world of plants (Wen et al. 2009; Hawes et al. 2011). ET formation relies on common cellular and molecular mechanisms from vertebrates to invertebrates.

In conclusion, the knowledge about the production of ETs in invertebrates confirms that the extracellular release of chromatin is an ancient defense process, and has been conserved through evolution.
